# Supplementary material for: Uncovering the spin ordering in magic-angle graphene via edge state equilibration
Source: Nat Commun. 2024 May 21;15:4321. doi: 10.1038/s41467-024-48385-z (PMC11109299; doi:10.1038/s41467-024-48385-z)
Supplement: Supplementary file 1 — Supplementary Information [file 41467_2024_48385_MOESM1_ESM.pdf]

Supplementary Information for:
"Uncovering the spin ordering in magic-angle graphene via edge state equilibration"

Jesse C. Hoke,[1,2,3] Yifan Li,[1,2,3] Julian May-Mann,[1,4] Kenji Watanabe,[5] Takashi Taniguchi,[6] Barry Bradlyn,[4] Taylor L. Hughes,[4] and Benjamin E. Feldman[1,2,3,*]

[1] *Department of Physics, Stanford University, Stanford, CA 94305, USA*
[2] *Geballe Laboratory for Advanced Materials, Stanford, CA 94305, USA*
[3] *Stanford Institute for Materials and Energy Sciences, SLAC National Accelerator Laboratory, Menlo Park, CA 94025, USA*
[4] *Department of Physics and Institute for Condensed Matter Theory, University of Illinois at Urbana-Champaign, Urbana, IL 61801, USA*
[5] *Research Center for Electronic and Optical Materials, National Institute for Materials Science, 1-1 Namiki, Tsukuba 305-0044, Japan*
[6] *Research Center for Materials Nanoarchitectonics, National Institute for Materials Science, 1-1 Namiki, Tsukuba 305-0044, Japan*

1. SUPPLEMENTARY FIGURES

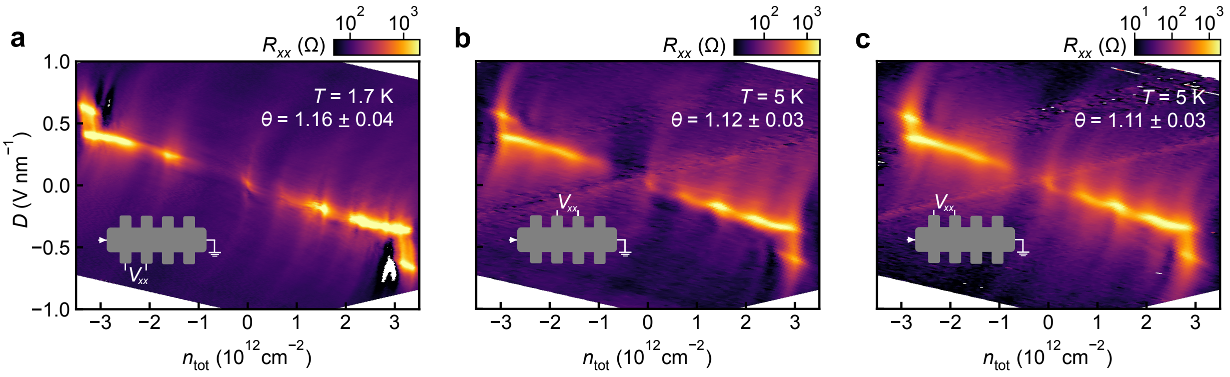

FIG. Supplementary Figure 1: **Sample homogeneity**. Longitudinal resistance R_{xx} as a function of total carrier density n_{tot} and displacement field D for three different contact pairs separate from the one discussed in the main text.

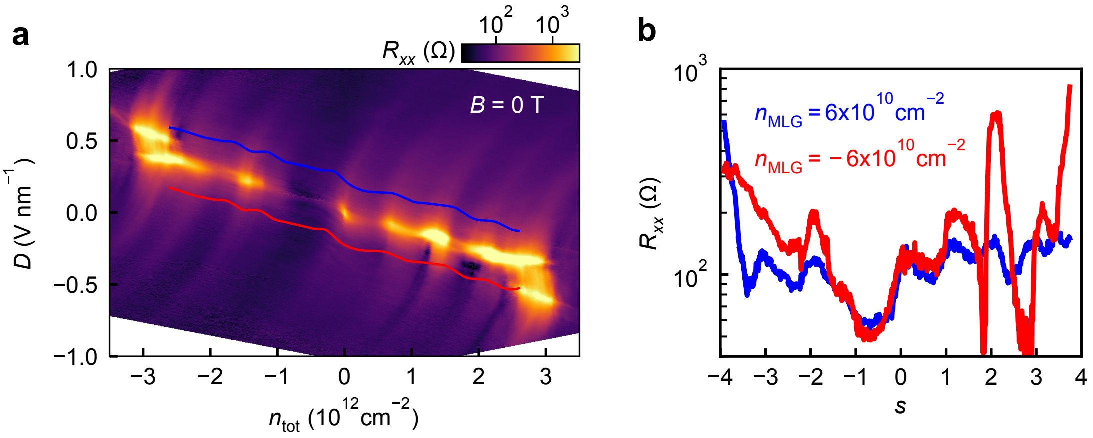

FIG. Supplementary Figure 2: **Resistance of MATBG at non-zero density of the monolayer graphene**. **a**, R_{xx} as a function of n_{tot} and D at temperature $T = 1.7$ K and magnetic field $B = 0$ T. Blue and red curves represent where the monolayer graphene is at fixed $n_{\mathrm{MLG}} = \pm 6 \times 10^{10}$ cm^{-2}, respectively. **b**, R_{xx} as a function of moiré filling factor s along the blue and red curves in **a**.

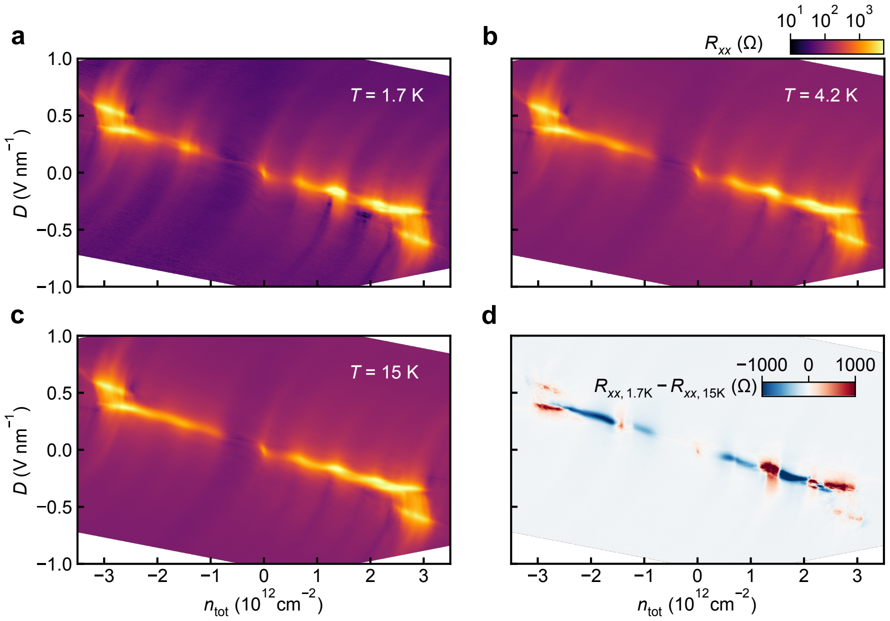

FIG. Supplementary Figure 3: **Temperature dependence at zero magnetic field**. **a**, **b**, **c**, R_{xx} as a function of n_{tot} and D at temperature T = 1.7 K, 4.2 K and 15 K, respectively. The appearance of a resistive peak near moiré filling factor s = -1 at higher temperatures reflects a Pomeranchuk-like effect in magic-angle twisted bilayer graphene (MATBG). **d**, $R_{xx,1.7\mathrm{K}} - R_{xx,15\mathrm{K}}$ as a function of n_{tot} and D, revealing insulating temperature dependence at $s = \pm 2, 3$, and ± 4.

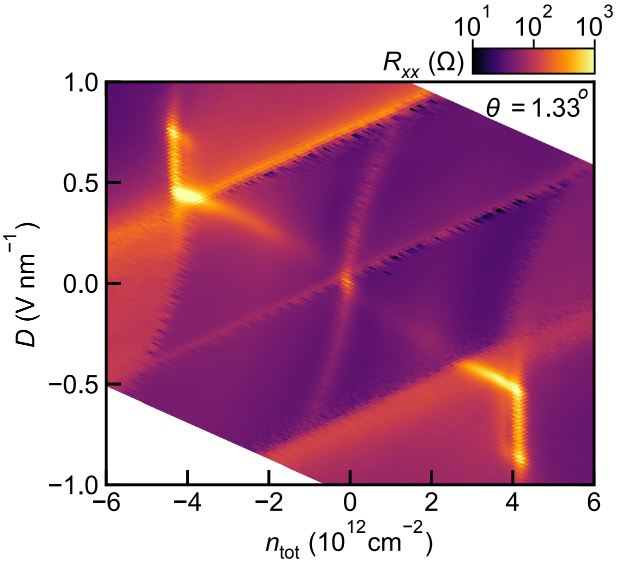

FIG. Supplementary Figure 4: **Transport of second device**. R_{xx} as a function of n_{tot} and D at T = 5 K for a second twist-decoupled device. Based on transport (see Methods), we estimate a twist angle of $\theta \approx 1.33°$ between the near-aligned layers that form its moiré subsystem. An analysis of an optical image (see Methods) of the stack indicates the twist angle between the moiré subsystem and the monolayer graphene subsystem is approximately 15°. Resistive peaks appear at $s = 0$ and ± 4 when the decoupled graphene monolayer is at its charge neutrality point. An S-shaped resistive feature near $n_{tot} = 0$ extends towards higher displacement fields as the graphene carrier density is adjusted while maintaining a fixed density of the moiré subsystem. This feature also displays the expected Dirac scaling $\mu_{\mathrm{MLG}} \propto \mathrm{sgn}(n_{\mathrm{MLG}})|n_{\mathrm{MLG}}|^{1/2}$. This phenomenology is consistent with the primary device reported in the main text. The straight diagonal features (which are independent of V_t) are due to parallel transport in a portion of the device that is only bottom gated.

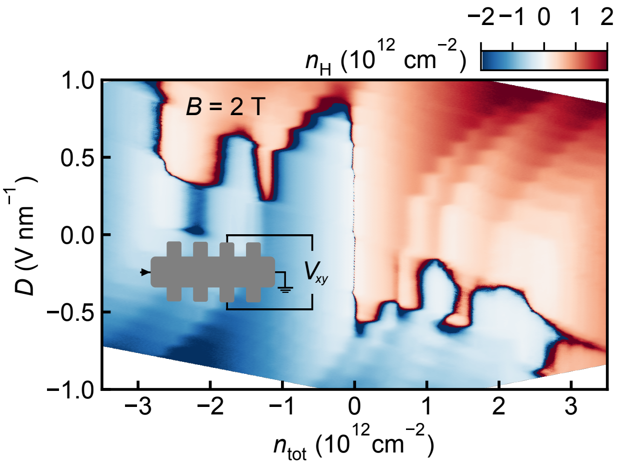

FIG. Supplementary Figure 5: **Hall density at** $B = 2$ **T**. Hall density n_{H} as a function of n_{tot} and D at $B = 2$ T. The Hall density qualitatively matches what is expected for the combined system, with resets in the effective MATBG carrier density occurring near certain integer s.

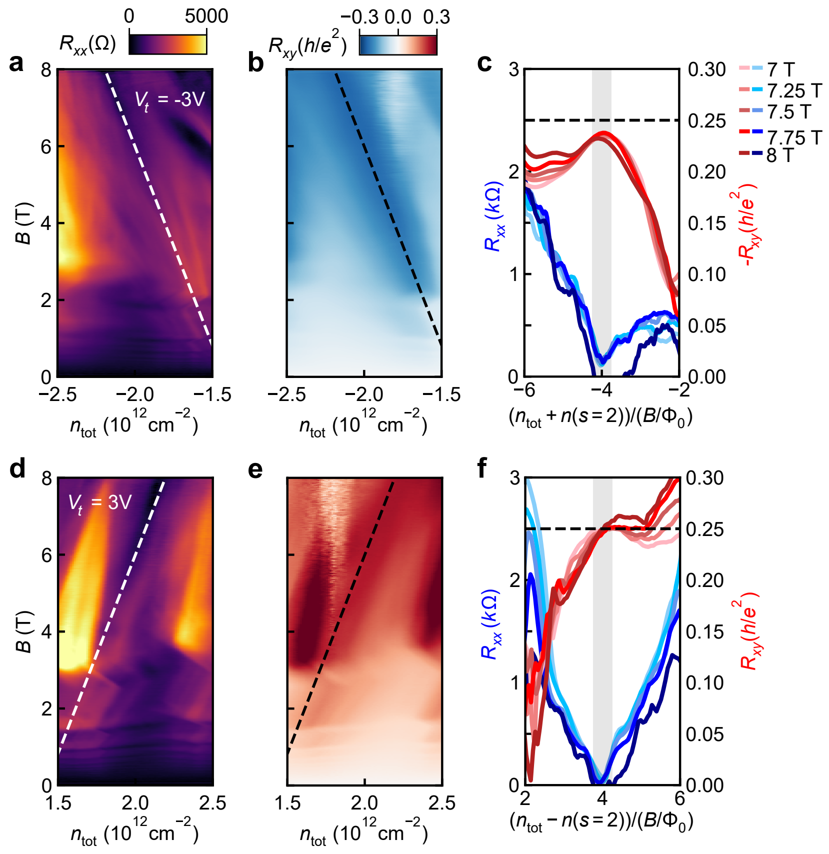

FIG. Supplementary Figure 6: **Landau fan measurement of even Chern insulators in the co-propagating regime**. **a**, **b**, R_{xx} and R_{xy} as a function of n_{tot} and B at fixed top gate voltage $V_t = -3$ V near the $(t, s) = (-2, -2)$ Chern insulator (ChI), where t is the Chern number. **c**, Line cuts of R_{xx} and R_{xy} showing well developed minima near zero R_{xx} and near-quantized R_{xy} for the $\nu_{\mathrm{MLG}} = -2/(t, s) = (-2, -2)$ state. **d**, **e**, **f**, Same as **a**, **b**, **c**, but for the $\nu_{\mathrm{MLG}} = 2/(t, s) = (2, 2)$ state when $V_t = 3$. Data are identical to that presented in Fig. 3, but zoom-ins and line cuts of the relevant features are shown here for clarity. Data collected at $T \approx 300$ mK.

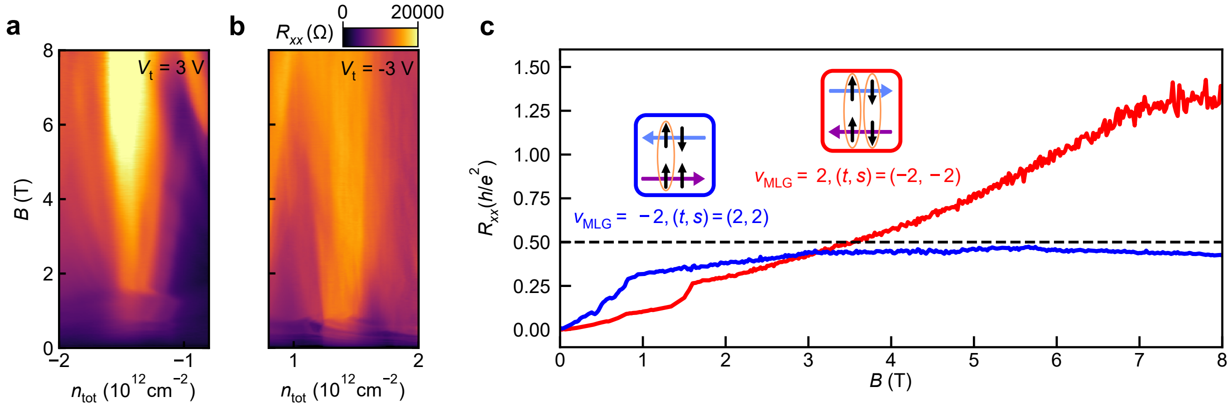

FIG. Supplementary Figure 7: **Landau fan measurement of even Chern insulators in the counter-propogating regime**. **a**, **b**, R_{xx} as a function of n_{tot} and B at fixed $V_t = 3$ V and $V_t = -3$ V, respectively, near the $\nu_{\mathrm{MLG}} = \pm 2/(t,s) = (\mp 2, \mp 2)$ states. **c**, R_{xx} as a function B for the $\nu_{\mathrm{MLG}} = 2/(t,s) = (-2,-2)$ (red) and $\nu_{\mathrm{MLG}} = -2/(t,s) = (2,2)$ (blue) states. Data are identical to that presented in Fig. 3, but zoom-ins and line cuts of the relevant features are shown here for clarity. Data collected at $T \approx 300$ mK.

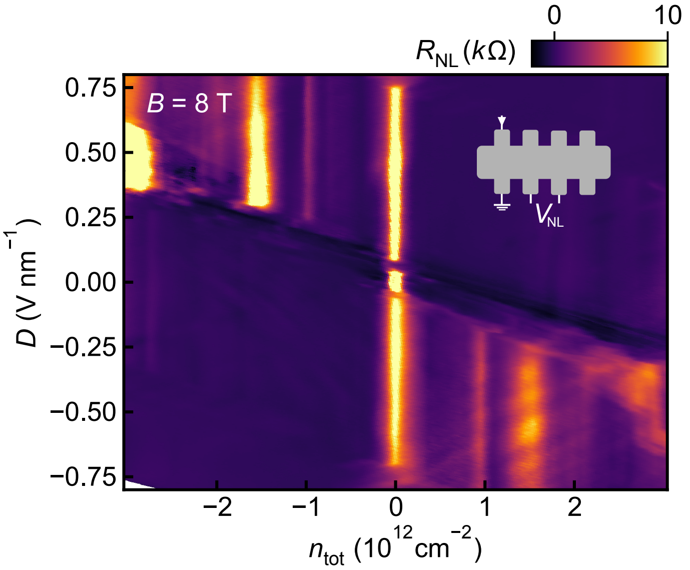

FIG. Supplementary Figure 8: **Non-local transport** Non-local resistance R_{NL} measured using the contact configuration shown in the inset as a function of n_{tot} and D at $B = 8$ T.

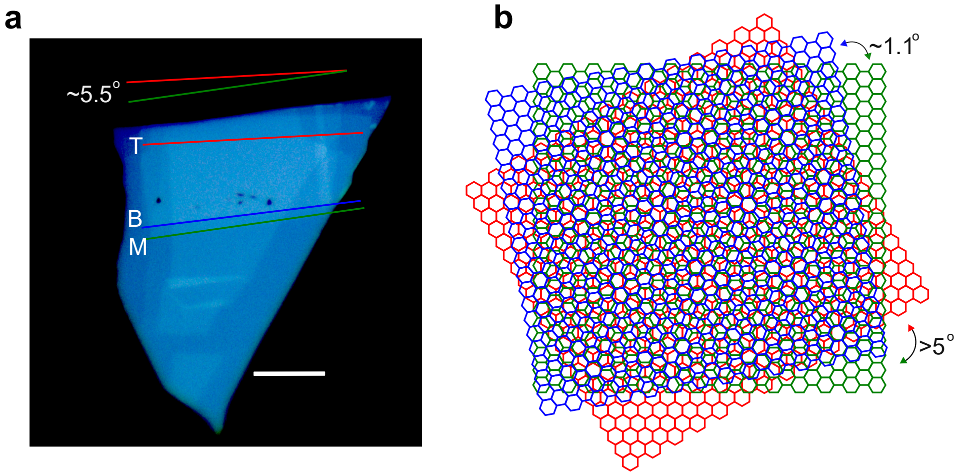

FIG. Supplementary Figure 9: **Twist angle between graphene layers**. **a**, Optical image of the trilayer stack after being picked up with an hBN flake on the polymer stamp but before deposition onto the bottom gate. Red, green, and blue lines are drawn on top of the AFM cut edges of the individual top, middle, and bottom monolayer flakes, respectively. From this optical image we estimate a twist angle between the top and middle layer of $\sim 5-6°$. The white scale bar is 10 μm. **b**, Schematic representation of the 3 graphene layers and their relative twists. Interlayer angles are exaggerated in the image for clarity.

2. SUPPLEMENTARY NOTE: CHEMICAL POTENTIAL EXTRACTION AND INTERLAYER CAPACITANCE ESTIMATE

To extract the chemical potentials of the MLG and MATBG subsystems, we use a model based on the screening of electric fields similar to that in Supplementary Refs. [1–4]. Although there is no hBN spacer between the MLG and the MATBG in our device, the large twist angle between these subsystems electronically decouples them so that a similar analysis applies and we can model the two subsystems with an interlayer capacitance C_i. This model yields the following equations for the densities n_i and chemical potentials μ_i of a given subsystem i when the other remains at charge neutrality:

$$n_{\mathrm{MLG}} = C_t V_t/e + (C_t + C_i)C_b V_b/eC_i, \tag{1}$$

$$n_{\mathrm{MATBG}} = C_b V_b/e + (C_b + C_i)C_t V_t/eC_i, \tag{2}$$

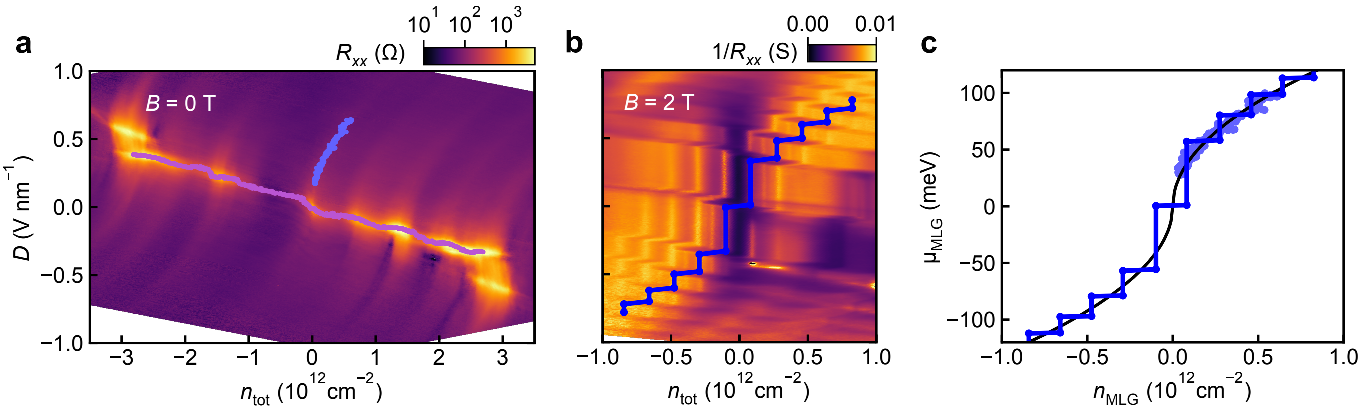

FIG. Supplementary Figure 10: **Extraction of the MLG and MATBG chemical potentials, and interlayer capacitance**. **a**, Longitudinal resistance R_{xx} as a function of total carrier density n_{tot} and displacement field D at zero magnetic field B with the extracted R_{xx} maxima corresponding to where $\mu_{\mathrm{MATBG}} = 0$ (light blue) and $\mu_{\mathrm{MLG}} = 0$ (light purple). **b**, $1/R_{xx}$ as a function of n_{tot} and D at $B = 2$ T. The dark blue curve tracks the maxima of $1/R_{xx}$ where $\mu_{\mathrm{MATBG}} = 0$. **c**, Extracted MLG chemical potentials μ_{MLG} at $B = 0$ (light blue) and $B = 2$ T (dark blue) using the points in **a** and **b**. A least squares fit of the data to the theoretically predicted functional form of the graphene chemical potential at $B = 0$ is shown in black.

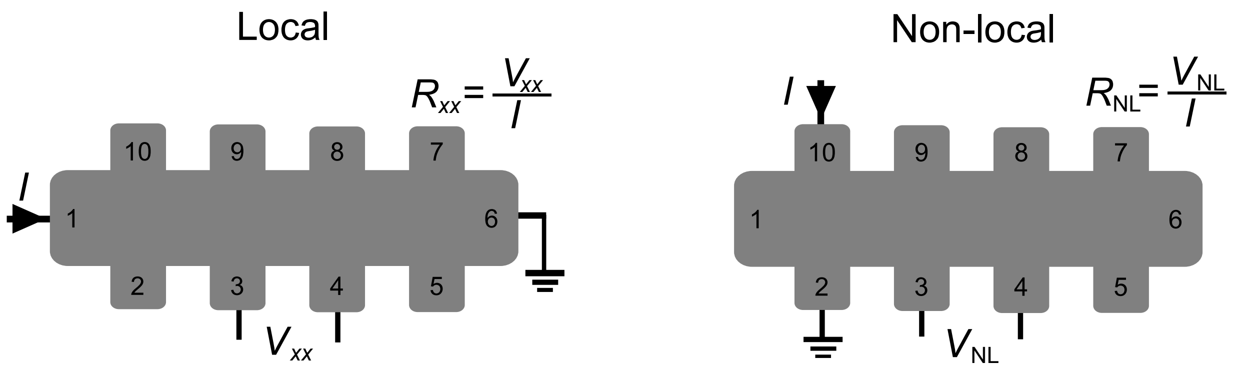

FIG. Supplementary Figure 11: **Resistance measurement geometries**. Local (left) and non-local (right) resistance measurement geometries. Contacts are labeled 1-10.

$$\mu_{\mathrm{MLG}} = -eC_bV_b/C_i, \tag{3}$$

$$\mu_{\mathrm{MATBG}} = -eC_tV_t/C_i \tag{4}$$

Here, e is the electron charge, and $C_{b(t)}$ and $V_{b(t)}$ are respectively the geometric capacitance and voltage applied to the bottom (top) gate. In the above equations, C_i is an unknown. However, because $\mu_{\mathrm{MLG}} = \mathrm{sgn}(n_{\mathrm{MLG}})\hbar v_F(\pi|n_{\mathrm{MLG}}|)^{1/2}$, C_i can be used as a fitting parameter to Supplementary equation 3 and experimentally determined ($\hbar$ is the reduced Planck's constant and we take the graphene Fermi velocity $v_F = 1.12 \times 10^6$ m/s). This value for C_i can then be used in Supplementary equation 4 to obtain $\mu_{\mathrm{MATBG}}(n_{\mathrm{MATBG}})$. To find C_i, we use the locations of the maxima in longitudinal resistance R_{xx} along $\mu_{\mathrm{MATBG}} = 0$ (light blue points, Supplementary Figure 10**a**) and use a least squares fitting procedure to match them to the theoretically expected $\mu_{\mathrm{MLG}}(n_{\mathrm{MLG}})$ (black curve, Supplementary Figure 10**c**), yielding a best fit $C_i = 5.24 \pm 0.52$ μF cm^{-2}. This is consistent with previous estimates in other twisted graphene systems, falling between the values extracted for twisted bilayer graphene and twisted double bilayer graphene [5–7]; while we cannot rule out that there is some hybridization between layers which renormalizes the Fermi velocity of the MLG subsystem [8], the agreement supports the validity of assuming a bare graphene Fermi velocity $v_F = 1.12 \times 10^6$ m/s.

A similar procedure can be used to extract $\mu_{\mathrm{MLG}}(n_{\mathrm{MLG}})$ at nonzero perpendicular magnetic field B. In Supplementary Figure 10**b** we plot $1/R_{xx}$ at $B = 2$ T, where clear Landau level (LL) crossings are observed. Overlaid in dark blue are lines which track $n_{\mathrm{MATBG}} = 0$. The resulting MLG chemical potential extracted from the points in Supplementary Figure 10**b** is displayed in Supplementary Figure 10**c**.

To determine $\mu_{\mathrm{MATBG}}(n_{\mathrm{MATBG}})$, we track the R_{xx} maxima along $\mu_{\mathrm{MLG}} = 0$, which is overlaid in light purple in Supplementary Figure 10**a**. We then use Supplementary equation 4 to determine $\mu_{\mathrm{MATBG}}(n_{\mathrm{MATBG}})$, which is shown in Fig. 1**g** of the main text. The corresponding change in $\mu_{\mathrm{MATBG}}(n_{\mathrm{MATBG}})$ across the flat bands is $\delta\mu \sim 40$ meV, comparable in magnitude to previous reports of MATBG [2, 9–12], further justifying the assumption of decoupled subsystems and the use of the bare graphene Fermi velocity.

3. SUPPLEMENTARY NOTE: LANDAUER-BÜTTIKER ANALYSIS

For a system where the transport is dominated by edge modes, the Landauer-Büttiker formula establishes the relationship between the edge mode transport and the measured resistance for arbitrary contact configurations. For the device studied here (Supplementary Figure 11) we consider a sample with N edge modes coupled to 10 leads. Generically, the total transmission amplitude T_{nm} between the m-th and n-th contacts of a sample is given by

$$T_{nm} = \sum_{\alpha,\beta=1}^{N} |t_{m\alpha,n\beta}|^2, \tag{5}$$

where $|t_{m\alpha,n\beta}|^2$ is the amplitude for the α-th mode from terminal m to tunnel into the β-th mode at terminal n. Here $|t_{m\alpha,n\beta}|^2 = 1$ for perfect transmission and $|t_{m\alpha,n\beta}|^2 = 0$ for no transmission.

	$N_L = N_R = 1$	$N_L = N_R = 2$
$\tau = 0$	$R_{xx} = h/2e^2$, $R_{\rm NL} = h/5e^2$	$R_{xx} = h/4e^2$, $R_{\rm NL} = h/10e^2$
$\tau = 1/2$	—	$R_{xx} = h/2e^2$, $R_{\rm NL} = h/5e^2$
$\tau = 1$	$R_{xx} = R_{\rm NL} = \infty$	$R_{xx} = R_{\rm NL} = \infty$

TABLE Supplementary Table 1: **Landauer-Büttiker formula predictions**. Predicted resistance values based on the Landauer-Büttiker formula (Supplementary equation 7) for different numbers of left/right propagating edge modes (N_L and N_R) and scattering strengths τ. We only list values corresponding to $\tau = M/N_R$, where $M \leq N_R$ is an integer.

The Landauer-Büttiker formula relates the transmission amplitudes to the current at the m-th terminal, I_m, and the voltage at the n-th terminal, V_n, via

$$I_m = \frac{e^2}{h}(NV_m - \sum_n T_{mn}V_n), \tag{6}$$

where the e^2/h prefactor comes from the universal conductance of 1D channels. For perfect transmission in the case where the N edge modes consist of N_R right moving modes and N_L left moving modes, we have $T_{m,m+1} = N_R$ and $T_{m,m-1} = N_L$, while all other transmission amplitudes vanish. However, we can also consider the situation where the transmission is reduced by some amount of backscattering between the right and left moving edge modes. Assuming $N_R \geq N_L$ without loss of generality, we introduce a phenomenological parameter $0 \leq \tau \leq 1$ to parameterize the degree of backscattering. Physically, $\tau = 0$ indicates that there is no backscattering and $\tau = 1$ indicates complete backscattering, where the N_L left moving modes fully scatter off the right moving modes, removing all left moving modes, and leaving $N_R - N_L$ right moving modes. There are special cases where rational values of the form $\tau = M/N_L$ ($0 < M < N_L$) can occur if M of the N_L left moving modes are fully backscattered by the right moving modes, leading to effectively $N_R - M$ right moving modes and $N_L - M$ left moving modes. In principle, such values of τ could also arise from a situation in which more than M left moving modes partially scatter off the right moving modes, but obtaining those exact rational values of τ would require fine tuning. In general, other rational values and any irrational values of τ are necessarily fine tuned, and correspond to partial scattering of left and right moving modes.

The phenomenological parameter τ enters the Landauer-Büttiker formula via the transmission amplitudes $T_{m,m+1} = N_R - \tau N_L$ and $T_{m,m-1} = (1-\tau)N_L$. To calculate the expected R_{xx}, we substitute these transmission amplitudes into the Landauer-Büttiker formula (Supplementary equation 6). For our measurements we set the source-lead current I_1 to $I_1 = I$, and the drain-lead current I_6 to $I_6 = -I$, and all other currents to zero (Supplementary Figure 11). The resistance $R_{xx} \equiv \Delta V/I$, where ΔV is the voltage difference across the probe leads 3 and 4, is found to be:

$$R_{xx} = \frac{h}{e^2}\frac{(N_R - \tau N_L)(1-\tau)N_L}{(N_R - \tau N_L)^3 + ((1-\tau)N_L)^3}. \tag{7}$$

A similar derivation can be made for the non-local measurement geometries we use. For this geometry we set the source-lead current I_{10} to $I_{10} = I$, and the drain-lead current I_2 to $I_2 = -I$, and all other currents to zero (Supplementary Figure 11). The resistance $R_{\rm NL} \equiv \Delta V/I$, where ΔV is the voltage difference across the probe leads 3 and 4, is

$$R_{\rm NL} = \frac{h}{e^2}\frac{N_L N_R^6}{N_R^8 + N_R^6 N_L^2 + N_R^4 N_L^4 + N_R^2 N_L^6 + N_L^8} \tag{8}$$

when $\tau = 0$. For $\tau > 0$, $R_{\rm NL}$ is related to the above calculation by substituting $N_R \to N_R - \tau N_L$ and $N_L \to (1-\tau)N_L$.

We next discuss the predicted values of R_{xx} and $R_{\rm NL}$ for various cases directly relevant to what is studied in the main text. At $\nu_{\rm MLG}/\nu_{\rm MATBG} = \pm 1/\mp 1$, we have $N_L = N_R = 1$ and Supplementary equations 7 and 8 simplify to:

$$R_{xx} = \frac{h}{2e^2}\frac{1}{(1-\tau)}, \quad R_{\rm NL} = \frac{h}{5e^2}\frac{1}{(1-\tau)}. \tag{9}$$

If we assume τ can take only values 0 or 1, then either R_{xx}, $R_{\rm NL} = h/2e^2$, $h/5e^2$ or R_{xx}, $R_{\rm NL} \to \infty, \infty$, respectively. Our data are consistent with $\tau \sim 0$.

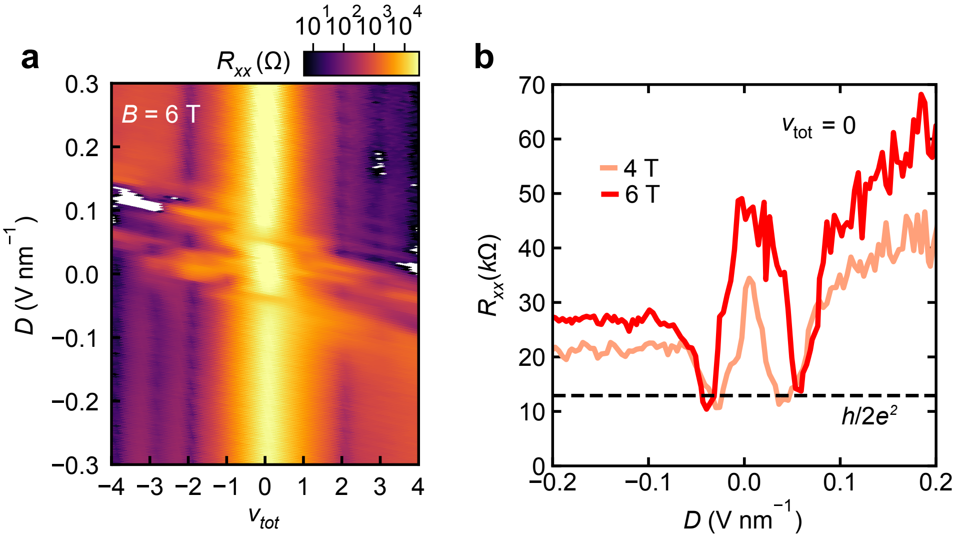

FIG. Supplementary Figure 12: **Edge state equilibration near the CNP of a second contact pair**. **a**, R_{xx} as a function of the total filling factor $\nu_{\rm tot} = \nu_{\rm MLG} + \nu_{\rm MATBG}$ and D at $B = 6$ T near the CNP. **b**, R_{xx} at $\nu_{\rm tot} = 0$ as a function of D (extracted from **a**). The data agree closely with observations from the contact pair discussed in the main text.

We are also interested in the case where $N_L = N_R = 2$, which is relevant for both the $\nu_{\rm MLG}/\nu_{\rm MATBG} = \pm 2/\mp 2$ and $\nu_{\rm MLG} = \pm 2/(t,s) = (\mp 2, \mp 2)$ states. The former correspond to quantum Hall states emanating from the charge neutrality point (CNP) in MATBG, and the latter to Chern insulators (ChIs) in MATBG, where t is the Chern number and s is the zero-field intercept. In this case Supplementary equations 7 and 8 become:

$$R_{xx} = \frac{h}{4e^2}\frac{1}{(1-\tau)}, \qquad R_{\rm NL} = \frac{h}{10e^2}\frac{1}{(1-\tau)}. \tag{10}$$

If we now assume τ can take only the rational values 0, 1/2, or 1, then either R_{xx}, $R_{\rm NL} = h/4e^2$, $h/10e^2$, R_{xx}, $R_{\rm NL} = h/2e^2$, $h/5e^2$, or R_{xx}, $R_{\rm NL} \to \infty$, ∞ respectively. Our data for the $\nu_{\rm MLG}/\nu_{\rm MATBG} = \pm 2/\mp 2$ and $\nu_{\rm MLG} = 2/(t,s) = (-2,-2)$ state are most consistent with $\tau \sim 1$. In constrast, the $\nu_{\rm MLG} = -2/(t,s) = (2,2)$ behavior is most consistent with $\tau \sim 1/2$, i.e., there is one pair of edge states that backscatter and another pair of counterpropagating modes that remain decoupled. In all cases, the transverse resistance R_{xy} is zero. The predicted resistances for both R_{xx} and $R_{\rm NL}$ for $N_L = N_R = 1, 2$ and $\tau = 0, 1/2$, or 1 are summarized in Supplementary Table 1.

4. SUPPLEMENTARY NOTE: EDGE STATE EQUILIBRATION IN A SECOND CONTACT PAIR

In this section, we present additional measurements (Figs. Supplementary Figure 12 - Supplementary Figure 14) from a second pair of contacts (2 and 3 in Supplementary Figure 11) that exhibit qualitatively similar behavior and therefore largely corroborate the conclusions about spin polarization discussed in the main text. For this second contact pair, R_{xx} as a function of $\nu_{\rm tot}$ and D at $B = 6$ T (Supplementary Figure 12**a**) matches closely to the observations from the contact pair discussed in the main text (3 and 4 in Supplementary Figure 11). Specifically, at $\nu_{\rm MLG}/\nu_{\rm MATBG} = \pm 1/\mp 1$, local minima occur in R_{xx} that are near the expected quantized value for a single pair of counter-propagating edge modes of opposite spin (Supplementary Figure 12**b**).

Likewise, there is again an abrupt increase in R_{xx} in this second pair as $|D|$ increases and the system enters the regime where $\nu_{\rm MLG}/\nu_{\rm MATBG} = \pm 2/\mp 2$. The large R_{xx} well above $h/2e^2$ indicates backscattering between both pairs of edge modes and therefore that the even-integer broken-symmetry quantum Hall states near the CNP in MATBG are spin unpolarized.

We next discuss the behavior of the ChIs in the second contact pair (Supplementary Figure 13), focusing first on the co-propagating regime when $\nu_{\rm MLG} = \pm 2$. Similar to the contact pair studied in the main text, the $(t,s) = (-2,-2)$ ChI displays R_{xx} minima near zero that follow a slope with a total Chern number of -4 (due to the contribution from

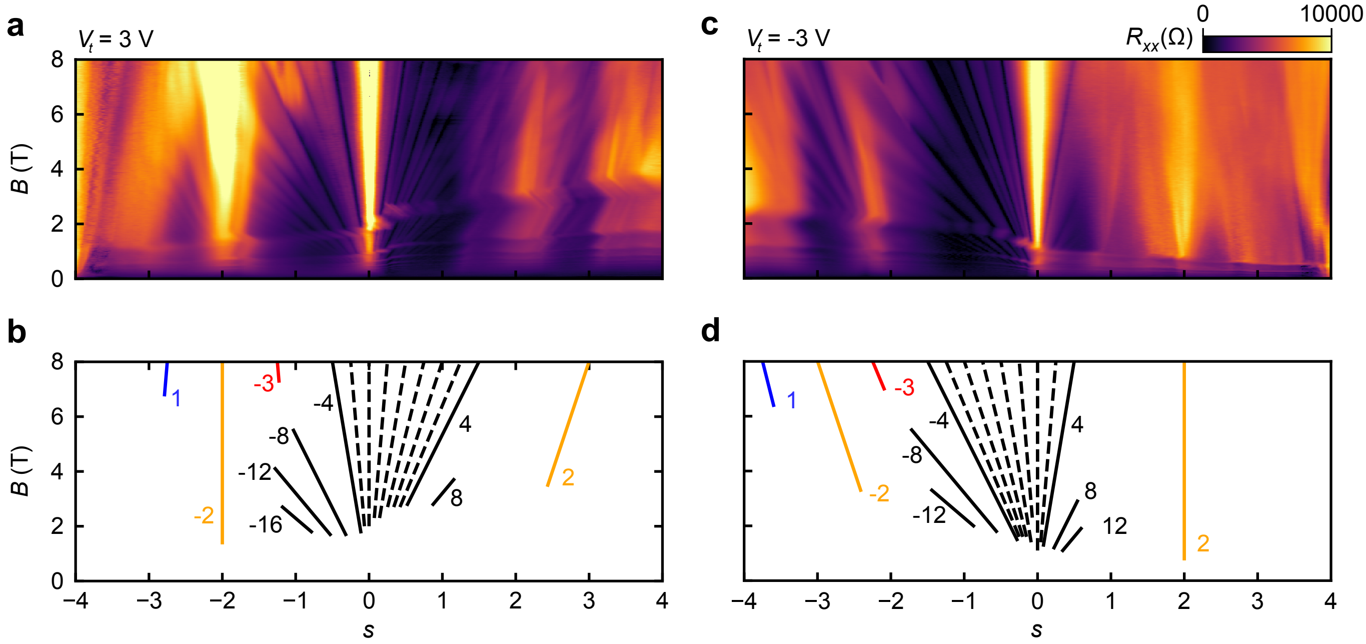

FIG. Supplementary Figure 13: **Landau fan measurement of a second contact pair**. **a**, R_{xx} as a function of s and B at fixed top gate voltage $V_t = 3$ V. **b**, Wannier diagram indicating the strongest quantum Hall and ChI states determined from **a**. The Chern numbers t of the MATBG states are labeled. At high fields, the total Chern numbers of each state are offset by 2 because $\nu_{\text{MLG}} = 2$. Black, red, orange, and blue lines correspond to states with zero-field intercepts $s = 0$, $s = |1|$, $s = |2|$, and $s = |3|$, respectively. For states with $s = 0$, $t \equiv \nu_{\text{MATBG}}$. Black dashed lines label the MATBG symmetry broken quantum Hall states $-4 < \nu_{\text{MATBG}} < 4$. **c**, **d**, Same as **a**, **b**, but for $V_t = -3$, where $\nu_{\text{MLG}} = -2$ at high fields. Data collected at $T \approx 300$ mK.

$\nu_{\text{MLG}} = -2$) in the co-propgating regime (Supplementary Figure 14**a,b**). In contrast, the $(t, s) = (2, 2)$ ChI is less well formed in the co-propagating regime: the R_{xx} minima are not as pronounced and their exact positions do not always perfectly fall along a single linear trajectory with a total Chern number of 4, as would be expected when accounting for the contribution from $\nu_{\text{MLG}} = 2$ (Supplementary Figure 14**c,d**).

In the counter-propagating regime with $\nu_{\text{MLG}} = 2$, the $(t, s) = (-2, -2)$ state displays high $R_{xx} > h/2e^2$ that increases with B, particularly above 5 T where the minima in R_{xx} become well-developed (Supplementary Figure 14**e,g**). This matches what is observed in the contact pair described in the main text, further supporting the conclusion that the $(t, s) = (-2, -2)$ ChI is spin unpolarized. In contrast, when $\nu_{\text{MLG}} = -2$ the $(t, s) = (2, 2)$ ChI displays a much smaller R_{xx} and weaker B-dependence (Supplementary Figure 14**f,g**). The reduced R_{xx} relative to the hole-doped side of MATBG is similar in some ways to the results presented in the main text. Importantly, however, the R_{xx} of this contact pair is not quantized to $h/2e^2$; instead, the resistance is lower than $h/4e^2$, the expected value for two fully decoupled counter-propagating edge modes. This may in part reflect that the $(t, s) = (2, 2)$ ChI is itself less well formed, as is evident from its behavior in the co-propagating regime (Supplementary Figure 14**c**,**d**). If the $(t, s) = (2, 2)$ state is not fully gapped or is more spatially inhomogeneous, this would likely lead to additional bulk contributions from the metallic-like character of the MATBG subsystem and therefore could ultimately lead to decreased resistance. For completeness, we also mention that it is possible that edge states with equal spin are not well coupled for this contact pair on the electron side, particularly given the generic observation of lower resistance for spin-unpolarized broken-symmetry states near the CNP when the MATBG is electron doped. However, we do not have an explanation for why this would be the case, nor why it would be more pronounced for this contact pair.

5. SUPPLEMENTARY NOTE: DISCUSSION OF ALTERNATE EXPLANATIONS AND QUANTITATIVE RESISTANCE VALUES

In the main text, we highlight general features of the resistance observed in the counter-propagating edge mode regime and their most natural qualitative interpretations. In this section, we provide further quantitative discussion and address alternative potential explanations for the data in more detail.

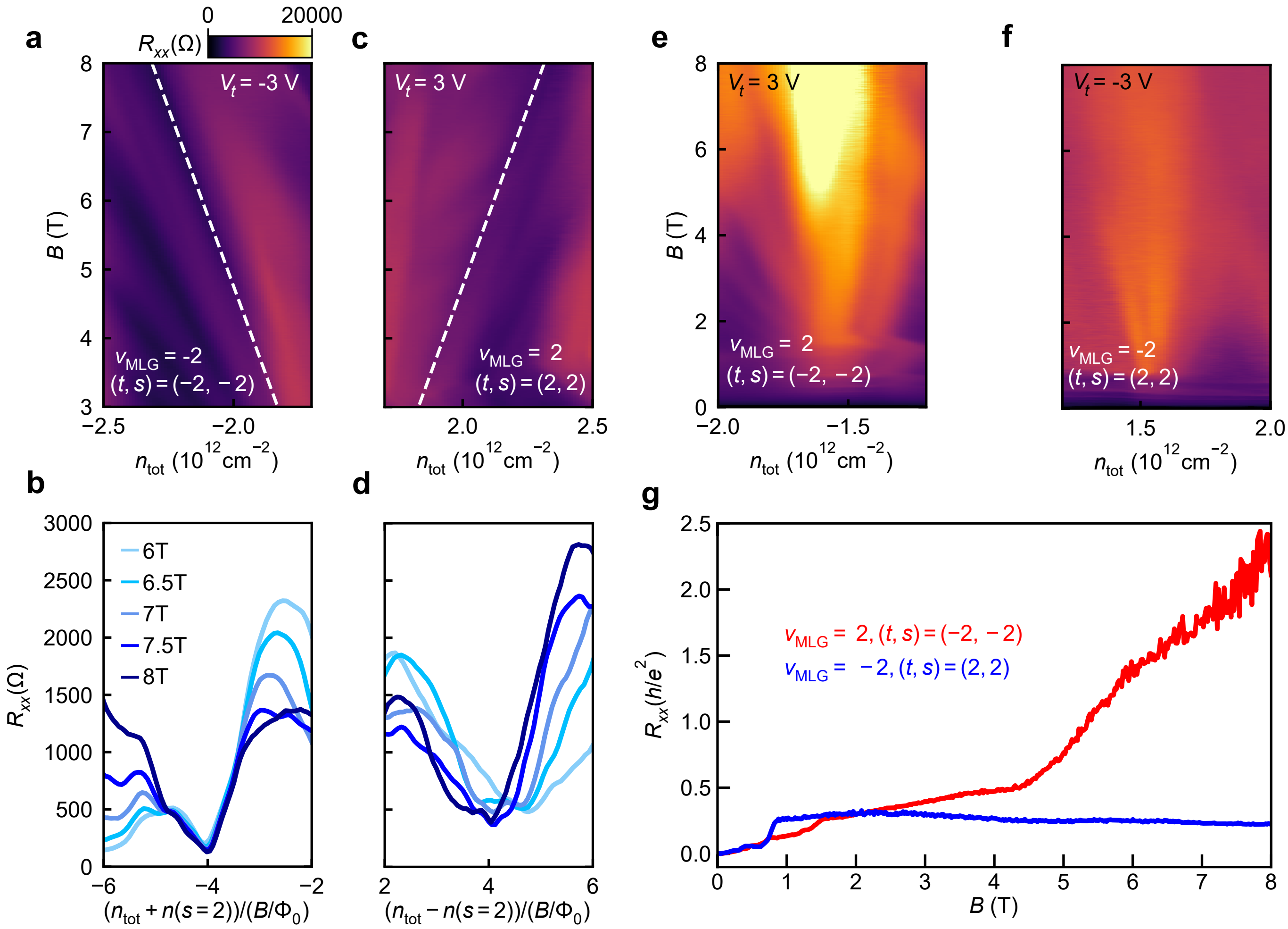

FIG. Supplementary Figure 14: **Edge state equilibration of the even Chern insulators for a second contact pair**. **a**, R_{xx} as a function of n_{tot} and B for a second contact pair near the $(t, s) = (-2, -2)$ ChI in the co-propagating regime. **b**, R_{xx} line cuts of the $\nu_{\text{MLG}} = -2/(t, s) = (-2, -2)$ state at different B forming well defined minima near zero. **c**, **d**, Same as **a**, **b**, but for the $\nu_{\text{MLG}} = 2/(t, s) = (2, 2)$ in the co-propogating regime. Minima in R_{xx} are less well developed. **e**, **f**, R_{xx} as a function of n_{tot} and B for the second contact pair near the $(t, s) = (\mp 2, \mp 2)$ ChIs, respectively, in the counter-propagating regime. **g**, R_{xx} as a function of B for the $\nu_{\text{MLG}} = \pm 2/(t, s) = (\mp 2, \mp 2)$ states. Data are identical to that presented in Supplementary Figure 13, but zoom-ins and line cuts of the relevant features are shown here for clarity. Data collected at $T \approx 300$ mK.

a. Resistances at $\nu_{\text{MLG}}/\nu_{\text{MATBG}} = \pm 1/\mp 1$

We focus first on the behavior at $\nu_{\text{MLG}}/\nu_{\text{MATBG}} = \pm 1/\mp 1$. The observed longitudinal and non-local resistances are close to their expected quantized values for a pair of decoupled counter-propagating edge modes, but they do exhibit deviations toward lower resistance, especially at the highest magnetic fields. Below, we discuss potential causes for such discrepancies and why the interpretation of opposite-spin counter-propagating edge states remains the most natural explanation for the data.

There is evidence of quantum Hall states and edge mode transport in both MATBG and MLG subsystems at the relevant fillings. For example, we observe local minima in R_{xx} in the co-propagating regime when $\nu_{\text{MLG}}/\nu_{\text{MATBG}} = \pm 2/\pm 1$, both in measurements at fixed B (Supplementary Figure 15 and Supplementary Figure 12**a**) and in Landau fans at fixed V_t (Fig. 3 and Supplementary Figure 13). We also observe local minima in R_{xx} when $\nu_{\text{MLG}}/\nu_{\text{MATBG}} = \pm 1/0$ (Supplementary Figure 15). These observations indicate developing MATBG and MLG quantum Hall states at $\nu_{\text{MATBG}} = \pm 1$ and $\nu_{\text{MLG}} = \pm 1$. Furthermore, as discussed in the main text, the appreciable R_{NL} at $\nu_{\text{MLG}}/\nu_{\text{MATBG}} = \pm 1/\mp 1$ demonstrates the presence of counter-propagating edge modes. Likewise, we observe steps in chemical potential which provide additional thermodynamic evidence for a bulk gap in the MLG subsystem at $\nu_{\text{MLG}} = \pm 1$. These manifest as features in R_{xx} that persist at constant filling over a range in displacement field; their extent in

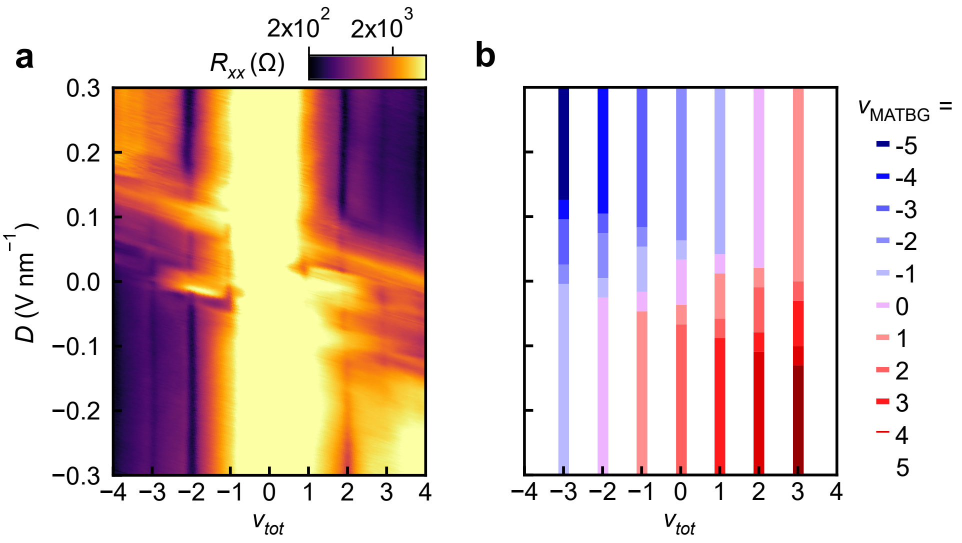

FIG. Supplementary Figure 15: **Transport of the MATBG-MLG quantum Hall bilayer**. **a**, R_{xx} as a function of $\nu_{\rm tot}$ and D at $B = 8$ T near the CNP. The data are the same as presented in Fig. 2**a** in the main text, but are reproduced here for clarity. **b**, Schematic map of the possible combined MATBG and MLG quantum Hall filling factors. Each color represents a specific $\nu_{\rm MATBG}$ in the MATBG subsystem. Clear minima in R_{xx} are, for example, observed at $\nu_{\rm MLG}/\nu_{\rm MATBG} = \pm 2/\pm 1$ and $\nu_{\rm MLG}/\nu_{\rm MATBG} = \pm 1/0$, which are signatures of quantum Hall states $\nu_{\rm MATBG} = \pm 1$ and $\nu_{\rm MLG} = \pm 1$, respectively.

D is a proxy for the size of the gap at $\nu_{\rm MLG} = \pm 1$. Well-formed features are evident at $\nu_{\rm MLG} = \pm 1$ (see, e.g., Supplementary Figure 12 and Supplementary Figure 15).

Despite the above signatures consistent with quantum Hall states at the relevant fillings, we do not measure perfectly quantized resistance. One way that $R_{xx} < h/2e^2$ could arise is if there is some parallel bulk conduction (e.g. if the quantum Hall states are not fully developed or if the device is inhomogeneous). We note that at $\nu_{\rm MLG}/\nu_{\rm MATBG} = 0/0$, R_{xx} is on the order of 40 - 60 kΩ, which can be used as an estimate of residual bulk conduction because no edge modes are expected there. A resistance of 40 - 60 kΩ in parallel with the expected $h/2e^2 = 12.9$ kΩ edge mode resistance would give a total resistance of 9.8 - 10.6 kΩ. These values are consistent with those that we observe, suggesting that additional weak bulk conduction could reasonably explain the small deviations in R_{xx} below $h/2e^2$.

Effects of parallel bulk conduction between contacts should be avoidable by measuring resistance in a non-local geometry, which is sensitive only to edge contributions. Indeed, at $\nu_{\rm MLG}/\nu_{\rm MATBG} = -1/1$, we observe that $R_{\rm NL}$ exhibits a plateau-like feature very close to $h/5e^2$, the predicted quantized value for this geometry. However, at $\nu_{\rm MLG}/\nu_{\rm MATBG} = 1/-1$, $R_{\rm NL}$ drops below $h/5e^2$. One likely contributing factor for this dip in $R_{\rm NL}$ is an artifact due to regions of the contacts to the device that are top gated but not bottom gated. Specifically, we observe a diagonal feature with negative $R_{\rm NL}$ that is approximately parallel to the bottom gate voltage V_b axis (Supplementary Figure 8) and intersects the $\nu_{\rm MLG}/\nu_{\rm MATBG} = 1/-1$ state (but *not* the $\nu_{\rm MLG}/\nu_{\rm MATBG} = -1/1$ state). The correlation of this feature with V_b suggests that it arises in a portion of the contact that is not bottom gated. This may spuriously lower the measured $R_{\rm NL}$ of the $\nu_{\rm MLG}/\nu_{\rm MATBG} = 1/-1$ state. Additionally, it is possible the lower $R_{\rm NL}$ may in part be due to 'leakage' of the edge current into alternate current paths through the bulk that do not connect the probed pair of contacts, i.e. that terminate at a different contact.

Given the above evidence for incipient quantum Hall states at the relevant fillings, signatures of edge conductance in non-local resistance, and measured resistance which approaches the quantized values expected for decoupled counter-propagating edge modes (and potential explanations for deviations) for multiple contact pairs, we conclude that the most likely explanation is that the MLG and MATBG states have opposite spin polarization at $\nu_{\rm MLG}/\nu_{\rm MATBG} = \pm 1/\mp 1$. This is also consistent with expectations for spin polarization based on the Zeeman effect.

b. Resistances at $\nu_{\rm MLG}/\nu_{\rm MATBG} = \pm 2/\mp 2$ and $\nu_{\rm MLG} = 2/(t,s) = (-2,-2)$

In the counter-propagating regime where $\nu_{\rm MLG}/\nu_{\rm MATBG} = \pm 2/\mp 2$, as well as when $\nu_{\rm MLG} = 2$ and $(t,s) = (-2,-2)$, the measured R_{xx} and $R_{\rm NL}$ both exceed the corresponding quantized values expected for a single decoupled pair of counter-propagating edge modes. As noted in the main text, this indicates that there is backscattering between both

pairs of counter-propagating modes and therefore that the MATBG state is spin-unpolarized in all of those cases. However, especially when the MATBG is electron doped, the measured resistances do not exceed the quantized values by a very large factor. Therefore, we discuss alternative potential explanations for the data below.

One possibility to consider is whether sample inhomogeneity could lead to disorder-induced equilibration of edge modes even if they have opposite spins. If there are multiple segments (separated by disordered regions) between contacts, each with a quantized resistance, they would add in series and could increase the measured resistance beyond a quantized value [13–15]. We view this scenario as highly unlikely for several reasons. First, there is relatively low twist angle disorder (Supplementary Figure 1), and we observe qualitatively similar behavior for multiple contact pairs as well as in non-local measurements. Second, although the maximum resistance only reaches a few times the quantized value, it is an appreciable fraction of the resistance at $n_{\mathrm{tot}} = 0$ and $D = 0$, where both MLG and MATBG are in broken-symmetry states at $\nu = 0$, and no bulk conductance or edge modes are expected. Finally, we observe close to quantized values when $\nu_{\mathrm{MLG}}/\nu_{\mathrm{MATBG}} = \pm1/\mp1$, indicating a decoupled pair of edge modes. There is no obvious reason to expect that these would not also be similarly affected by disorder along the edge. Thus, the abrupt jump in resistance from the $\nu_{\mathrm{MLG}}/\nu_{\mathrm{MATBG}} = \pm1/\mp1$ states to the $\nu_{\mathrm{MLG}}/\nu_{\mathrm{MATBG}} = \pm2/\mp2$ states provides strong evidence for significantly enhanced backscattering between the edge modes in the latter case.

It is also worth commenting on the fact that R_{xx} is lower for the $\nu_{\mathrm{MLG}}/\nu_{\mathrm{MATBG}} = 2/-2$ state than the $\nu_{\mathrm{MLG}}/\nu_{\mathrm{MATBG}} = -2/2$ state, particularly when measured using contacts 3 and 4 (the primary contact pair studied in the main text). Despite this difference, our data still suggest that both topological sectors host two pairs of counter-propagating spin unpolarized edge modes and that the edge modes can efficiently back scatter off each other. This conclusion is motivated by the fact that the R_{NL} of the $\nu_{\mathrm{MLG}}/\nu_{\mathrm{MATBG}} = 2/-2$ state (which should be primarily determined by edge mode transport) is more than twice as large as the expected R_{NL} of a system with a single counter-propagating pair of edge modes ($h/5e^2$ in this geometry). Additionally, the value of R_{NL} is comparable between the $\nu_{\mathrm{MLG}}/\nu_{\mathrm{MATBG}} = 2/-2$ and $\nu_{\mathrm{MLG}}/\nu_{\mathrm{MATBG}} = -2/2$ state. This indicates that the relatively lower R_{xx} of the $\nu_{\mathrm{MLG}}/\nu_{\mathrm{MATBG}} = 2/-2$ state is likely due to weak parallel bulk conduction, possibly caused by a difference in the size of the gaps of these states due to electron-hole asymmetry in MATBG. We additionally note that the R_{xx} for the second contact pair discussed in Section 3 displays $R_{xx} > h/e^2$ for both $\nu_{\mathrm{MLG}}/\nu_{\mathrm{MATBG}} = \pm2/\mp2$, and better quantized $R_{xx} = h/2e^2$ at $\nu_{\mathrm{MLG}}/\nu_{\mathrm{MATBG}} = \pm1/\mp1$, suggesting that this region of the device may be less susceptible to additional bulk contributions compared to the primary contact pair. Finally, recent calculations indicate the possibility of partial spin polarization of the broken-symmetry states due to interaction-driven renormalization of Hofstadter subbands [16]. If there is electron-hole asymmetry in the MATBG band structure and/or exchange interaction, this may affect the relative degree of spin polarization of the two states and therefore the inter-edge backscattering and measured resistances.

c. Resistance at $\nu_{\mathrm{MLG}} = -2$ and $(t, s) = (2, 2)$

Finally, we discuss in more detail alternate scenarios that could lead to the observed resistances of the $\nu_{\mathrm{MLG}} = -2$ and $(t, s) = (2, 2)$ state. The longitudinal and non-local resistances of this state exhibit plateaus that are nearly quantized at the values predicted by the Landauer-Büttiker formalism (Supplementary equation 9) for a single pair of counter-propagating edge modes. This is consistent with a picture where the state initially has two pairs of counter propagating edge modes, but efficient backscattering occurs only for one pair, resulting in a topological state having a single remaining pair of counter propagating edge modes (see Sec. 2 for further discussion). This would naturally occur if the $(t, s) = (2, 2)$ is spin-polarized: when combined with the spin-unpolarized edge modes of the $\nu_{\mathrm{MLG}} = -2$ state, this leads to a pair of counter-propagating modes having the same spin (that can efficiently backscatter) and one pair of counter-propagating modes having opposite spin that *cannot* efficiently scatter off each other (in the absence of magnetic impurities).

However, other mechanisms for decoupling are possible and could contribute to the lower resistances we observe (in all contact pairs) relative to the $\nu_{\mathrm{MLG}} = 2/(t, s) = (-2, -2)$ state. We discuss these possibilities below and the degree to which they are likely to be contributing factors.

One possibility to consider is that both the $\nu_{\mathrm{MLG}} = -2$ and $(t, s) = (2, 2)$ edge modes are spin unpolarized, but with weakened symmetry-allowed scattering processes. This is unlikely because such scattering processes appear to be robust for the other states we measure. The near-quantization of both R_{xx} and R_{NL} for the contacts discussed in the main text would also require fine-tuning, making this explanation less likely (though not impossible). This contrasts with the natural expectation for a spin-polarized state.

Another possibility is that the $(t, s) = (2, 2)$ is not well-developed, similar to what is observed for the second contact pair discussed above in Supplementary Note 4 of the supplement. This explanation, however, is not consistent

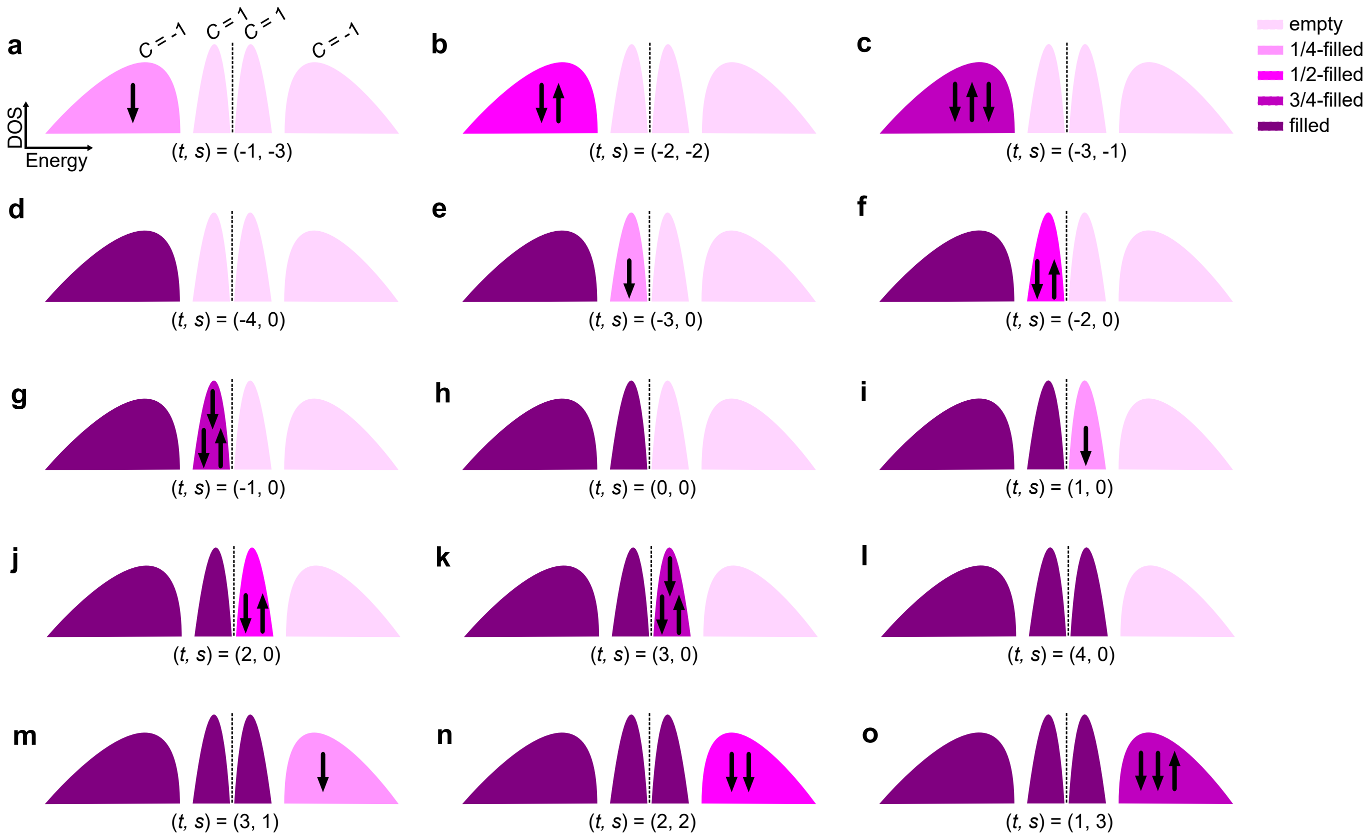

FIG. Supplementary Figure 16: **Spin filling sequence of the Hofstadter subbands**. Schematic representation of the Hofstadter subbands as they are filled with different spin states. The Chern numbers of each subband are labeled in the top left image, and the central dashed line marks the charge neutrality point. Note that these diagrams are meant only as a cartoon representation and that the relative heights and bandwidths do not reflect calculated values. The different colors represent different levels of filling for each of the subbands, as indicated in the legend. The arrows represent the inferred spin polarization of the flavors occupying the bands.

with the well-developed zeros in R_{xx} and quantized $R_{xy} = h/4e^2$ in the co-propagating regime for the primary pair contacts (Supplementary Figure 6). For these reasons, the R_{xx} and R_{NL} for the contact pair discussed in the main text suggests that the $(t, s) = (2, 2)$ ChI is most likely spin polarized.

6. SUPPLEMENTARY NOTE: INTERPRETATION OF RESULTS IN THE HOFSTADTER SUBBAND PICTURE

In this section, we discuss the inferred sequence of fillings of Hofstadter subbands based on our data. In a perpendicular magnetic field, MATBG states form a fractal Hofstadter energy spectrum [16–25], which is relevant to both the ChIs and the quantum Hall states emanating from the CNP. The spectrum is smeared out by temperature and disorder so that only the largest gaps between subbands are experimentally resolved [2, 26–28]. This leads to three especially prominent subbands with respective Chern numbers of -1, 2, and -1; there are four copies of this spectrum due to the four possible flavors from the spin and valley degrees of freedom in MATBG. Furthermore, the central $C = 2$ subband arises from the two Dirac crossings (per spin and valley flavor) at the MATBG CNP, i.e. the two moiré valleys. This central subband can be further split into two $C = 1$ subbands in the presence of moiré valley splitting, which can arise from heterostrain and/or interactions that break M_y symmetry. As discussed below, we observe evidence of such splitting and thus schematically illustrate a central doublet of $C = 1$ subbands in Supplementary Figure 16. The primary sequence of ChIs in MATBG (satisfying $|t + s| = 4$) is well described by sequentially filling the $C = -1$ bands with different particle flavors, while the quantum Hall states near the charge neutrality arise from a sequential filling of the two central $C = 1$ subbands. The total Chern number t of a given state in MATBG is the sum of the Chern numbers of all the filled subbands.

For topological phases of matter, the bulk-edge correspondence predicts that the net number of low-energy topologically protected edge states matches the bulk Chern number. Thus, like quantum Hall states, ChIs arising from occupation of Hofstadter subbands are also expected to exhibit an insulating bulk and chiral edge modes. As discussed in the main text, our experiments allow us to infer the spin polarization of these MATBG edge modes for both the quantum Hall states near the CNP and the ChIs. We can then infer the spin-resolved filling sequence of the topological Hofstadter subbands in MATBG from our data, which is schematically illustrated in Supplementary Figure 16.

As detailed in the main text, the data indicate that the spin polarization of the edge mode of $(t, s) = (-1, -3)$ is identical to that of $\nu_{\mathrm{MLG}} = 1$, which corresponds to just a single flavor occupying the lowest-energy $C = -1$ subband. Thus in Supplementary Figure 16**a**, we show the $(t, s) = (-1, -3)$ with a single spin-down electron occupying the the first $C = -1$ subband. For $(t, s) = (-2, -2)$, we observe the edge modes to be spin-unpolarized. Thus the next flavor to occupy the first $C = -1$ subband must be spin-up (Supplementary Figure 16**b**). This, together with Zeeman considerations, also allows us to deduce the spin polarization in the (-3,-1) and (-4,0) states (Supplementary Figure 16**c**,**d**), the latter of which is a flavor symmetric state (all four flavors filled).

Once the first $C = -1$ subband is fully filled (with 2 spin-down and 2 spin-up flavors), the central $C = 1$ subbands begin to be occupied. At $\nu_{\mathrm{MATBG}} = -2$ [equivalent to $(t, s) = (-2, 0)$], our data indicates spin unpolarized edge modes, which corresponds to one spin-up and spin-down flavor occupying the first $C = 1$ subband (Supplementary Figure 16**f**). Note that if the central subband was *not* split and instead retained its $C = 2$ Chern number, then exchange interactions would favor occupying it with a single flavor, which would lead to a spin-polarized state at $\nu_{\mathrm{MATBG}} = -2$. That we instead observe a spin-unpolarized state is evidence of moiré valley splitting and the formation of a $C = 1$ doublet of subbands. The same argument applies for the $\nu_{\mathrm{MATBG}} = 2$ state [equivalent to $(t, s) = (2, 0)$], where we also observe spin-unpolarized edge modes. Together with the spin identification based on counter-propagating opposite-spin states at $\nu_{\mathrm{MLG}}/\nu_{\mathrm{MATBG}} = \pm 1/\mp 1$ [equivalent to $(t, s) = (\pm 1, 0)$], and assuming the Zeeman effect dictates the spin of the $(\pm 3, 0)$ states, we determine the spin filling sequence of the central Hofstadter subbands in Supplementary Figure 16**e**-**l**.

Once the first three subbands are all filled, electrons begin filling the highest energy $C = -1$ subbands to form the ChIs on the electron side. Our data suggests that the $(t, s) = (2, 2)$ state is likely spin-polarized. Thus we tentatively illustrate this state with two spin-down electrons in the highest energy $C = -1$ subbands (spin-down assuming the Zeeman effect favors this over spin-up) (Supplementary Figure 16**n**). Note that although two additional spin-down flavors occupy the subband, the edge modes will be spin-up. This is because the additional two $C = -1$ spin-down edge modes will effectively "cancel" out the two $C = 1$ spin-down edge modes from the $(t, s) = (4, 0)$ state, leaving behind two remaining spin-up edge modes in the $(t, s) = (2, 2)$ state. This reasoning leads to the filling sequence for the uppermost $C = -1$ subband illustrated in (Supplementary Figure 16**m**-**o**).

7. SUPPLEMENTARY NOTE: DISCUSSION OF GENERALITY OF THE RESULTS

For most combinations of MLG/MATBG states, our data provide consistent results from multiple contact pairs and in measurements conducted in a non-local geometry. Furthermore, the features in the data that we ascribe to MATBG match well to previously observed generic behavior in MATBG devices. However, we acknowledge that the measurements presented here are primarily from one device, and it is possible that the spin polarization of correlated states could have some sample dependency. This is common in moiré systems such as MATBG, where microscopic details, such as strain and twist angle, can lead to different observed correlated ground states [29–34]. Even if such microscopic details modify device specific behavior, our work clearly demonstrates that symmetry breaking terms arising from exchange effects and strain are competitive with Zeeman splitting, and that even the preference for spin-polarized versus unpolarized ground states can exhibit electron-hole asymmetry. Importantly, the measurements also serve as a proof of principle demonstration of a new device architecture and a powerful approach to identify quantum degrees of freedom, applied to a sample whose features are consistent with prior reports in MATBG.

SUPPLEMENTARY REFERENCES

* Electronic address: bef@stanford.edu

[1] S. Kim, I. Jo, D. C. Dillen, D. A. Ferrer, B. Fallahazad, Z. Yao, S. K. Banerjee, and E. Tutuc, Physical Review Letters **108**, 116404 (2012), publisher: American Physical Society.
[2] J. M. Park, Y. Cao, K. Watanabe, T. Taniguchi, and P. Jarillo-Herrero, Nature **592**, 43 (2021), number: 7852.
[3] A. Mreńca-Kolasińska, P. Rickhaus, G. Zheng, K. Richter, T. Ihn, K. Ensslin, and M.-H. Liu, 2D Materials **9**, 025013 (2022), number: 2 Publisher: IOP Publishing.
[4] X. Liu, N. J. Zhang, K. Watanabe, T. Taniguchi, and J. I. A. Li, Nature Physics **18**, 522 (2022), number: 5 Publisher: Nature Publishing Group.
[5] J. D. Sanchez-Yamagishi, T. Taychatanapat, K. Watanabe, T. Taniguchi, A. Yacoby, and P. Jarillo-Herrero, Physical Review Letters **108**, 076601 (2012), number: 7.
[6] P. Rickhaus, M.-H. Liu, M. Kurpas, A. Kurzmann, Y. Lee, H. Overweg, M. Eich, R. Pisoni, T. Taniguchi, K. Watanabe, K. Richter, K. Ensslin, and T. Ihn, Science Advances **6**, eaay8409 (2020), number: 11.
[7] P. Rickhaus, G. Zheng, J. L. Lado, Y. Lee, A. Kurzmann, M. Eich, R. Pisoni, C. Tong, R. Garreis, C. Gold, M. Masseroni, T. Taniguchi, K. Wantanabe, T. Ihn, and K. Ensslin, Nano Letters **19**, 8821 (2019).
[8] A. Uri, S. C. de la Barrera, M. T. Randeria, D. Rodan-Legrain, T. Devakul, P. J. D. Crowley, N. Paul, K. Watanabe, T. Taniguchi, R. Lifshitz, L. Fu, R. C. Ashoori, and P. Jarillo-Herrero, Nature **620**, 762 (2023).
[9] J. Yu, B. A. Foutty, Y. H. Kwan, M. E. Barber, K. Watanabe, T. Taniguchi, Z.-X. Shen, S. A. Parameswaran, and B. E. Feldman, Nature Communications **14**, 6679 (2023).
[10] U. Zondiner, A. Rozen, D. Rodan-Legrain, Y. Cao, R. Queiroz, T. Taniguchi, K. Watanabe, Y. Oreg, F. von Oppen, A. Stern, E. Berg, P. Jarillo-Herrero, and S. Ilani, Nature **582**, 203 (2020), number: 7811.
[11] Y. Saito, F. Yang, J. Ge, X. Liu, T. Taniguchi, K. Watanabe, J. I. A. Li, E. Berg, and A. F. Young, Nature **592**, 220 (2021), number: 7853.
[12] D. Wong, K. P. Nuckolls, M. Oh, B. Lian, Y. Xie, S. Jeon, K. Watanabe, T. Taniguchi, B. A. Bernevig, and A. Yazdani, Nature **582**, 198 (2020), number: 7811.
[13] S. Wu, V. Fatemi, Q. D. Gibson, K. Watanabe, T. Taniguchi, R. J. Cava, and P. Jarillo-Herrero, Science **359**, 76 (2018).
[14] S. Essert and K. Richter, 2D Materials **2**, 024005 (2015).
[15] J. Maciejko, X.-L. Qi, and S.-C. Zhang, Phys. Rev. B **82**, 155310 (2010).
[16] X. Wang and O. Vafek, "Theory of correlated chern insulators in twisted bilayer graphene," (2023), arXiv:2310.15982 [cond-mat.mes-hall] .
[17] D. R. Hofstadter, Phys. Rev. B **14**, 2239 (1976).
[18] R. Bistritzer and A. H. MacDonald, Phys. Rev. B **84**, 035440 (2011).
[19] P. Moon and M. Koshino, Phys. Rev. B **85**, 195458 (2012).
[20] K. Hejazi, C. Liu, and L. Balents, Phys. Rev. B **100**, 035115 (2019).
[21] Y.-H. Zhang, H. C. Po, and T. Senthil, Phys. Rev. B **100**, 125104 (2019).
[22] B. Lian, F. Xie, and B. A. Bernevig, Phys. Rev. B **102**, 041402 (2020).
[23] X. Wang and O. Vafek, Phys. Rev. B **106**, L121111 (2022).
[24] J. Herzog-Arbeitman, A. Chew, D. K. Efetov, and B. A. Bernevig, Phys. Rev. Lett. **129**, 076401 (2022).
[25] D. Parker, P. Ledwith, E. Khalaf, T. Soejima, J. Hauschild, Y. Xie, A. Pierce, M. P. Zaletel, A. Yacoby, and A. Vishwanath, "Field-tuned and zero-field fractional chern insulators in magic angle graphene," (2021), arXiv:2112.13837 [cond-mat.str-el] .
[26] Y. Saito, J. Ge, L. Rademaker, K. Watanabe, T. Taniguchi, D. A. Abanin, and A. F. Young, Nature Physics **17**, 478 (2021), number: 4.
[27] J. Yu, B. A. Foutty, Z. Han, M. E. Barber, Y. Schattner, K. Watanabe, T. Taniguchi, P. Phillips, Z.-X. Shen, S. A. Kivelson, and B. E. Feldman, Nature Physics **18**, 825 (2022), number: 7 Publisher: Nature Publishing Group.
[28] Y. Choi, H. Kim, Y. Peng, A. Thomson, C. Lewandowski, R. Polski, Y. Zhang, H. S. Arora, K. Watanabe, T. Taniguchi, J. Alicea, and S. Nadj-Perge, Nature **589**, 536 (2021), number: 7843 Publisher: Nature Publishing Group.
[29] K. P. Nuckolls, R. L. Lee, M. Oh, D. Wong, T. Soejima, J. P. Hong, D. Călugăru, J. Herzog-Arbeitman, B. A. Bernevig, K. Watanabe, T. Taniguchi, N. Regnault, M. P. Zaletel, and A. Yazdani, Nature **620**, 525–532 (2023).
[30] Y. H. Kwan, G. Wagner, T. Soejima, M. P. Zaletel, S. H. Simon, S. A. Parameswaran, and N. Bultinck, Phys. Rev. X **11**, 041063 (2021).
[31] G. Wagner, Y. H. Kwan, N. Bultinck, S. H. Simon, and S. Parameswaran, Physical Review Letters **128** (2022), 10.1103/physrevlett.128.156401.
[32] S. Liu, E. Khalaf, J. Y. Lee, and A. Vishwanath, Physical Review Research **3** (2021), 10.1103/physrevresearch.3.013033.
[33] D. E. Parker, T. Soejima, J. Hauschild, M. P. Zaletel, and N. Bultinck, Phys. Rev. Lett. **127**, 027601 (2021).
[34] Z. Bi, N. F. Q. Yuan, and L. Fu, Phys. Rev. B **100**, 035448 (2019).
